# A Dielectric Elastomer-Based Multimodal Capacitive Sensor

**DOI:** 10.3390/s22020622

**Published:** 2022-01-14

**Authors:** Yuting Zhu, Tim Giffney, Kean Aw

**Affiliations:** 1 Department of Mechanical and Mechatronics Engineering, University of Auckland, Auckland 1010, New Zealand; y.zhu@auckland.ac.nz; 2 Department of Engineering Science, University of Auckland, Auckland 1010, New Zealand; 3 Department of Mechanical Engineering, University of Canterbury, Christchurch 8041, New Zealand; tim.giffney@canterbury.ac.nz

**Keywords:** dielectric elastomer, flexible pressure sensor, stretchable sensor, multi-location

## Abstract

Dielectric elastomer (DE) sensors have been widely used in a wide variety of applications, such as in robotic hands, wearable sensors, rehabilitation devices, etc. A unique dielectric elastomer-based multimodal capacitive sensor has been developed to quantify the pressure and the location of any touch simultaneously. This multimodal sensor is a soft, flexible, and stretchable dielectric elastomer (DE) capacitive pressure mat that is composed of a multi-layer soft and stretchy DE sensor. The top layer measures the applied pressure, while the underlying sensor array enables location identification. The sensor is placed on a passive elastomeric substrate in order to increase deformation and optimize the sensor’s sensitivity. This DE multimodal capacitive sensor, with pressure and localization capability, paves the way for further development with potential applications in bio-mechatronics technology and other humanoid devices. The sensor design could be useful for robotic and other applications, such as fruit picking or as a bio-instrument for the diabetic insole.

## 1. Introduction

Sensing is an essential function for the proprioception of any artificial system. The ability to measure force or pressure and map the force/pressure location increases the capability of these systems, including those applied to robotics. A sensor, with which the pressure can be quantified and the location can be mapped, can have many applications, such as object identification, wearable keyboard, etc. It can also be used, for example, as a clinical measurement mat to help foot exercise treatments for diabetic patients with ulcerated feet.

DE capacitive sensors have been used in many applications to detect pressure, strain, and touch. The capacitance changes when the sensing area is touched. In recent years, touch technology has become increasingly attractive for screen interfaces [1,2]. Jin et al. [3] used a dual-capacitor multi-sensor for their touch-curvature-pressure-strain sensing device. Other studies [4,5,6] have reported on touch sensing in tactile sensors for humanoid devices and artificial tactile applications. D’Souza et al. [7] show that most touch applications are targeted towards mobile devices and screen interfaces, but such applications are still at an early stage of development for soft wearable technology and humanoid devices.

In many applications, it is desirable to create sensors that combine more than one detection function in one device. This is referred to as multimodal. Many multimodal, or multifunctional, sensors have been developed [8,9,10,11] to measure parameters such as strain, pressure, and temperature, etc. Multimodal sensors have been developed and used as skin sensors for robotics applications, such as robotic grippers. For instance, Le et al. [9] developed a multimodal tactile sensor to measure the force on a Robotiq gripper, and Zhao et al. [12] used a multifunctional sensor for static and dynamic strain mapping by measuring the capacitance change, while Sun et al. [13] used current to identify touch/pressure. In our work, we have developed a sensor that can measure force/pressure and map the location of that force/pressure.

This soft multimodal capacitive sensor developed in this work aims to mimic the basic properties of human skin. The sensor should be soft and flexible, able to conform to the robotic curved body structures, detect light touch, and be easy and cheap to manufacture. Robotic skins have previously been developed, such as stretchable and wearable skin sensors that use polymer solar cells [14]. However, such devices using thin skin layers only would not suit soft robots without an optimal substrate due to lower sensitivity and the lack of substrate padding that could damage an object’s surface. Recently, capacitor sensor arrays for soft robotics using the multi-layered [15] technique have been developed, but the manufacturing process requires precision for each sensing element (tactel). Cannata et al. [16] used 12 capacitive tactels on a flexible substrate as artificial skin, capable of only measuring the contact pressure. Furthermore, Ohmura et al. [17] developed a tactile sensor skin that exhibited large hysteresis and is costly to make. Therefore, this study aimed to develop a soft multimodal sensor that mimics the human skin structure. The sensor should be soft and flexible, able to conform to the robotic curved body structures, detect light touch, and be easy and cheap to manufacture.

This study aimed to develop a DE sensor that can measure touch pressure and determine the touch location simultaneously. This sensor is a soft, flexible, and stretchable tactile DE multi-location capacitive sensor array with multiple touch locations. The touch pressure and location are measured by the capacitance variation resulting from the deformation of the sensor. This sensor module is composed of an array of two-dimensional capacitive sensors. The unique arrangement of the capacitive sensors layered atop each other, with and without overlap, leads to fewer capacitive sensors than the total number of touch locations (tactels). The aim of this paper is targeted for application where the location of force can be determined, such as the location of contact between the robotic gripper and the object. It is also suitable as a wearable/flexible keyboard.

## 2. Materials and Structures

**Figure 1:**
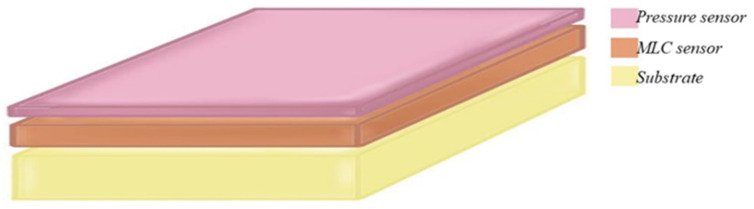
General cross-sectional layer structure of a multimodal DE.

The proposed multimodal sensor with a compliant substrate is illustrated in Figure 1. The prototype multimodal DE sensor, designed to have better deformability, has the following three main components: the pressure sensor (P) layer, MLC sensor layers, and a substrate layer. Furthermore, the sensor can also provide force feedback that would allow identification of the type of the object grasped, measure of the pressure, and tracking of the location during contact for specific applications.

The development of a unique MLC sensor using minimal pieces of overlapping capacitive DE sensors [18] in a shielded configuration is described here. The sensitivity of this sensor was limited since it was relatively thin and could not detect forces below 2 N without the addition of a PU soft substrate underneath it, as proven in prior research [19].

The DE sensor was chosen due to the silicone mechanical properties, which have a Young’s modulus of 0.55 MPa, and Poisson’s ratio of 0.49 falls within the properties of human skin [20]. The softness of the DE sensor can improve the contact quality between the object and manipulator and use the sense of touch to control the manipulator, a function for amputees.

### 2.1. Pressure Sensor Layer

The pressure sensor design used in our device is a shielded capacitive DE sensor containing two ground electrodes and one signal electrode. It is based on the designs described in the previous substrate study [19] but is fabricated with a much larger size for the multimodal sensor. The pressure sensor layer thickness was less than 1 mm, and it is added on top of the MLC sensor. The sensor design is robust against normal variations in layer thickness in the fabrication process and can be successfully fabricated by simple methods, such as hand-casting.

The pressure sensor (P) layer provides pressure information, i.e., the magnitude of the pressure through the amount of change in capacitance, and the tactel location through the MLC sensor underneath.

### 2.2. Multi-Location Capacitive DE Sensor (MLC) Layer Concept

**Figure 2:**
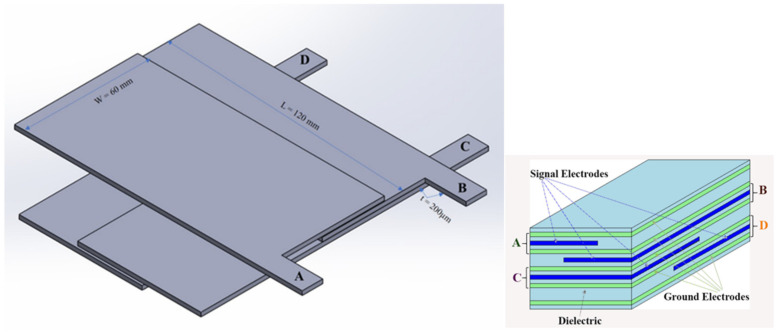
MLC sensor overview: MLC sensor layout with sensors of different sizes overlapping. A unique overlapping arrangement of 4 capacitive sensor arrangement to achieve 15 tactels.

In Figure 2, the basic concept for the new structure MLC, using a unique arrangement of overlapping capacitive sensors, is depicted. There are four pieces of overlapping capacitive sensors, A, B, C, and D, within the MLC sensor, which allows 15 tactels to be realized. This arrangement can be regarded as a 2 × 2 array arrangement as there are 2 sensor layers (sensors A and B) vertically overlapping each other and 2 sensor layers horizontally overlapping each other (sensors C and D). An MLC sensor made with four sensors, A, B, C, and D, which were laid out horizontally and vertically and then stacked over each other to form a unique overlapping arrangement. As this MLC has 15 tactels, the capability of this sensor and where a single touch on any part of this tactel can be identified, will be demonstrated.

Overall, this MLC sensor was fabricated with different sizes for sensors A, B, C, and D, with partial overlap, and arranged as a 2 × 2 arrangement, as shown in Figure 2.

The MLC sensor structure consists of nine layers of electrodes (Figure 2). Four of these are signal electrodes, and the other five are ground electrodes. Layers of silicone dielectric separate them. Sensor A is in the top layer, and sensor D is in the bottom layer. This means that sensor A will be deformed first when an object touches the sensor. The changes in sensor geometry due to deformation has been discussed in the previous substrate study [19]. When a force is applied, the deformation of the sensor is affected by the thickness of the different structural dielectric layers, and therefore the capacitance change in sensor A (top layer) should be slightly greater than that of the bottom layers.

**Figure 3:**
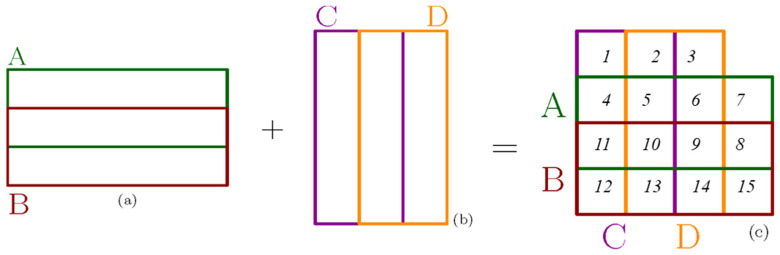
The arrangement for: (**a**) sensor elements A and B partially overlapped horizontally; (**b**) sensors C and D partially overlapped vertically, and (**c**) the final overlapping arrangement of the A/B and C/D sensors.

A unique overlapping layout for the four sensors as a 2 × 2 array is depicted in Figure 3, which is an illustrative view for a multi-capacitive sensor with two overlapping horizontal sensors, A and B (Figure 3a), two overlapping vertical sensors C and D (Figure 3b), and finally the unique arrangement of the four sensors (A, B, C, D) (Figure 3c).

As shown in Figure 3c, the final arrangement shows that the MLC sensor has partially overlapping areas of sensors A, B, C, and D in both horizontal and vertical directions.

**Figure 4:**
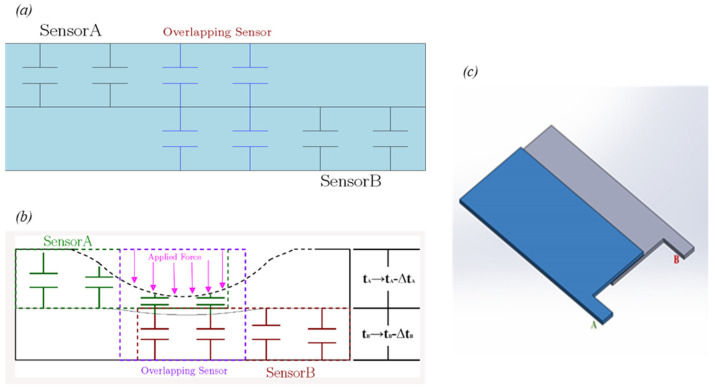
(**a**) Overlapping sensors A and B schematic view without contact; (**b**) Sensors’ capacitances change due to force applied. ∆t_A_—change in dielectric thickness of sensor element A; ∆t_B_—change in dielectric thickness of sensor B; (**c**) The view of sensors A and B with overlapped layers.

Figure 3 shows how the 4 sensor layers A, B, C, and D were overlapped to achieve 15 tactels. In Figure 4, the basic concept for the 2 pieces of overlapping capacitive sensors, A and B, is depicted. Figure 4b illustrates how the sensors’ capacitance changes are influenced by the touch position, the sensor deformation depth, and the force’s magnitude. If any tactel is pressed or touched, the capacitance of the sensors will increase because the thickness of the sensor reduces, and the area increases. Since the force is directly applied to sensor A (the top layer), this layer experiences more relative capacitance changes due to its greater change in thickness than sensor B as ∆t_A_ > ∆t_B,_ as illustrated in Figure 4b.

### 2.3. The Substrate of the Sensor

The substrate, an essential part of the multimodal sensor, is the interfacing layer between the sensor and the structure to which it is attached. The thickness of the substrate affects the sensitivity of the sensor. Our previous work [19] demonstrated that there is an optimized substrate thickness, and the optimization has been demonstrated to achieve a sensitivity of 2.7 pF/N.

### 2.4. The Multimodal Sensor

The multimodal capacitive sensor is the combination of three different layers (pressure, MLC, and substrate), described earlier. The MLC, with a 2 × 2 array, produces 15 tactels but with the addition of the P layer, a total of 16 tactels are realized. Tactel number 16 consist of only one sensor from the P layer.

**Figure 5:**
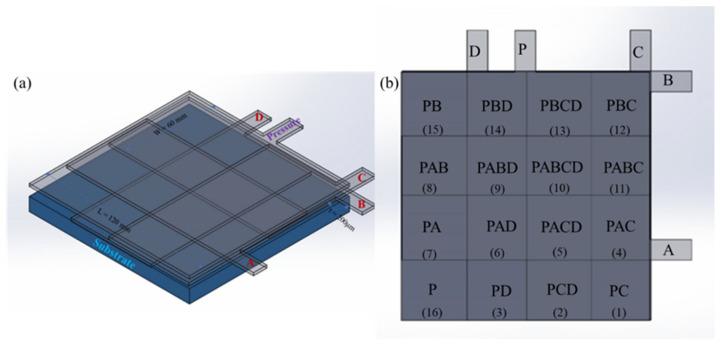
(**a**) A 3D view of the multimodal sensor showing the 5 sensor layers (P, A, B, C, and D); (**b**) a physical map of the 16 tactels.

Figure 5 shows the layers in the multimodal sensor. This multimodal sensor structure has five connections (P, A, B, C, and D) to a total of five pieces of DE capacitive sensors. In general, the sensors P, A, B, C, and D are used to locate the tactel that is touched, but it is P that allows the quantification of the touch pressure.

**Figure 6:**
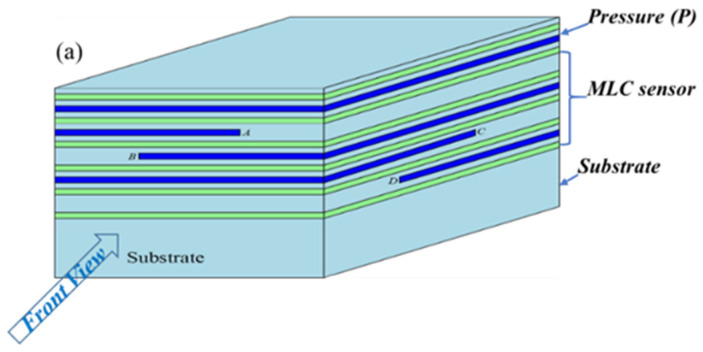

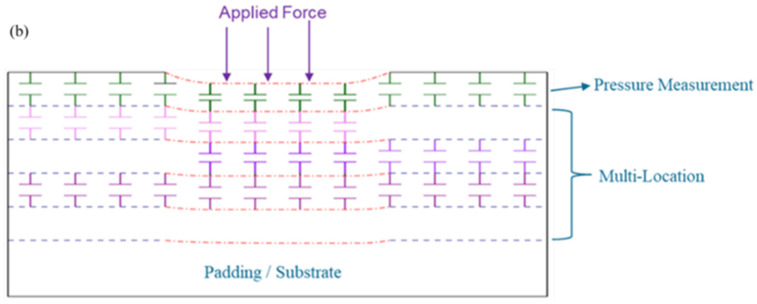
Multimodal capacitive DE sensor consisting of a pressure sensor, P (on the top of the sensor), MLC sensor (in the middle), and substrate/padding (bottom). Each sensor contains electrodes that are separated by a dielectric layer. (**a**) A cross-section of the sensor showing the different layers, (**b**) the change in capacitance in the different layer as viewed from the front.

The complete multimodal sensor structure, which is illustrated in the cross-section view of Figure 6, consists of the following three different layer groups: pressure sensor (P) layer, multi-location (MLC) sensor layer, and a substrate layer. The deformation of the top layer and the subsequent change in capacitance allows pressure measurement when a force is applied. When the sensor is loaded, as shown in Figure 6b, the pressure sensor (P) layer will be deformed, and the associated sensors (A, B, C, D) will also be deformed. Hence, the reading of the change in capacitance of the P sensor, together with the digital output of sensors A, B, C, and D of the MLC sensor, provide two pieces of information, i.e., the magnitude of the pressure through P and the tactel location through the digital output (A, B, C, D) of the MLC sensor. A substrate layer of optimum thickness [19] increases the sensor’s sensitivity and allows it to be sensitive to forces lower than 2 N. Hence, the layers of this multimodal sensor, from top to bottom, are the encapsulation (protection) layer of 100 μm, a pressure sensor layer, P (600 μm thick), the 4 sensors (A, B, C, and D) of the MLC sensor (total 2400 μm thick) in the middle, and the substrate (1900 μm thick) at the base.

## 3. Experimentations

The prototype sensors were fabricated for testing using a dielectric elastomer (DE). The sensors were hand-cast, with the sensor dielectric layers composed of StretchSense (Auckland, New Zealand) 270064 liquid silicone rubber (LSR) silicone (19 Shore A), and the sensor electrode layers were composed of StretchSense 270036 carbon-loaded silicone (pre-mixed by StretchSense). Due to commercial sensitivity, the exact composition could not be revealed.

The fabrication of the DE capacitive MLC sensor is as per the process introduced in Y. Zhu’s prior study [18]. After the MLC sensor was fabricated, a large DE pressure sensor (P) is added on top of the MLC sensor, consisting of four pieces of capacitive DE sensors (A, B, C, D) in an overlapping structure, leading to many tactels.

Each tactel needs to be calibrated before use, as the no-contact capacitances for each of the sensors, P, A, B, C, and D will be different. In this work, the proposed sensor was designed for forces between 0 and 10 N.

**Figure 7:**
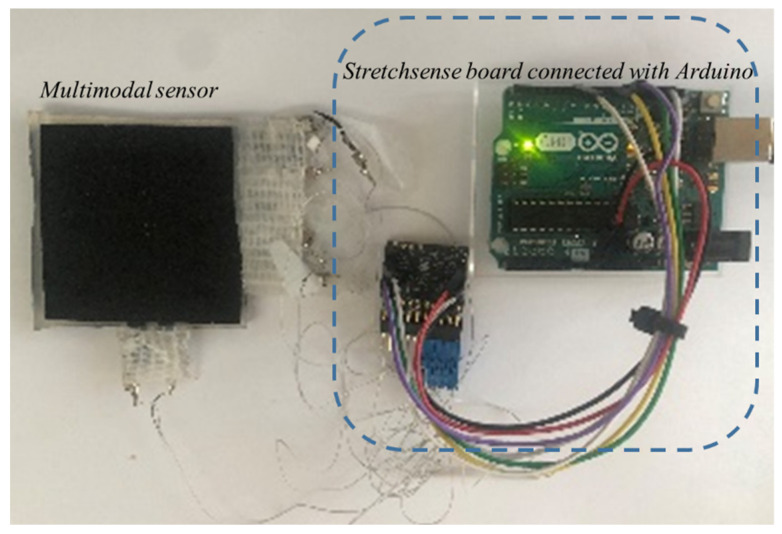
The electronics used to interface with the multimodal sensor.

The experimental setup for characterizing the fabricated sensors, where the sensor is connected to a Stretchsense© circuit board, converts the capacitance into the equivalent digital value (see Figure 7). The Stretchsense© board is connected to an Arduino that communicates with a computer where the LabVIEW program acquires and processes the data, displaying it graphically. This setup can display the sensor capacitance readings of each tactel.

For ease of quantifying the capacitance change within the five sensor layers (P, A, B, C, and D), despite the effects of stray capacitances, the no-contact capacitance was subtracted from each measured value in LabVIEW, and only the change in capacitance considered. Considering that the multimodal sensor has noise and parasitic effects, the threshold needs to be adjusted accordingly. When the sensor is touched/pressed, one or more of the five sensors will react with a change in the capacitance.

The LabVIEW program can indicate which tactel is touched/pressed through the reading of sensors P, A, B, C, and D (MLC sensor) based on the truth table in Table 1 and the amount of pressure is quantified with the amount of change in capacitance by sensor P. The adaptive thresholding is necessary to encode the change in capacitances into digital output, as the capacitance change will increase when the applied pressure is increased. The adaptive thresholding is simple and is adjusted by calculating the relative change in the P sensor to encode the capacitances value in P, A, B, C, and D into a digital value.

Table 1 is a truth table for each of the 16 tactels. When the multimodal sensor is not touched, there will not be any change in capacitance for P, A, B, C, and D sensors, which can be represented as PABCD = 00000. For example, when tactel # 4 is touched/pressed, the digital representation is PABCD = 11010. Furthermore, if tactel # 16 is touched/pressed, the digital output of PABCD = 10000. The amount of change in the capacitance of P can be used to quantify the magnitude of the touch force/pressure.

## 4. Results

**Figure 8:**
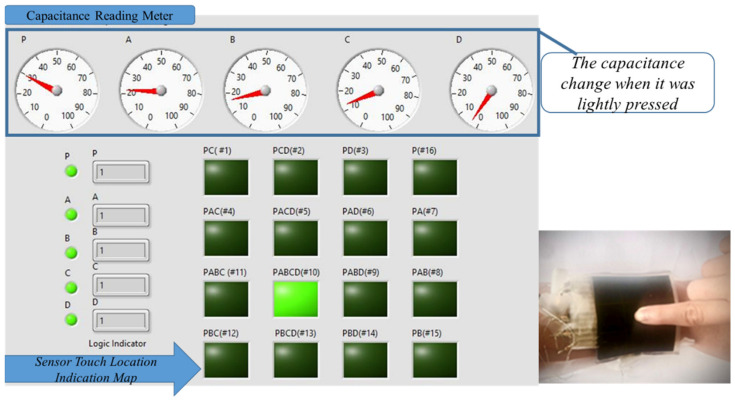
LabVIEW GUI readouts displaying multimodal sensor output in real-time when tactel # 10 was touched. The meter reads the capacitance change in the five sensors.

Figure 8 shows a multimodal sensor worn on the wrist, when tactel # 10 was pressed. Reading the capacitance meter shows that there were capacitance changes in P, A, B, and C. There was a very small change in the capacitance in D, but with adaptive thresholding applied, it can be converted into a digital sequence of PABCD = 11111. Referring to Table 1, this refers to tactel # 10. In Figure 8, the correct indicator lit up indicating that tactel # 10 was pressed.

**Figure 9:**
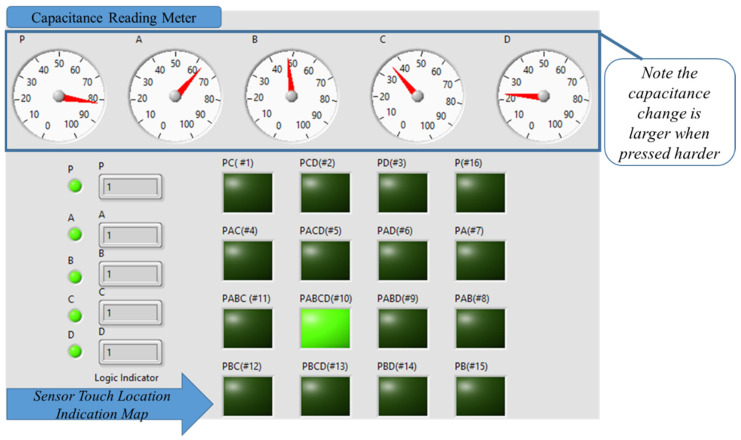
LabVIEW GUI readouts displayed real-time of the multi-functional sensor, when location # 10 pressed with greater force.

Figure 9 shows the same tactel (# 10) as in Figure 8 when it was pressed with a higher pressure. The change in capacitance reading for ‘P’ was much higher, as shown in Figure 9, than in Figure 8 when it was lightly pressed. Despite the higher change in the capacitance readings in P, A, B, C, and D, with adaptive thresholding, the digital reading of PABCD = 11111 is ensured for the identification of tactel # 10. Hence, it demonstrated the application as a soft wearable keyboard.

The dielectric spacing in the multimodal sensor is an important factor. The different thickness caused the sensitivity of each layer to be different. In this multimodal sensor, sensor P deformed more than the sensors A, B, C, and D in the MLC layer. This higher sensitivity is essential to quantify the magnitude of the pressure.

For example, when tactel # 4 was lightly touched when the sensor was worn around the wrist, and the LabVIEW GUI indicator lit up for this tactel with adaptive thresholding applied (PABCD = 11010), demonstrating its ability to identify the tactel correctly.

In another example, when tactel # 14 was pressed, the capacitance of P was much higher due to higher pressure applied, but with adaptive thresholding the digital value of PABCD = 10101 was encoded.

## 5. Discussion

A digital (binary) system can be used to identify the unique location of each tactel. Each tactel can be mapped with the unique combination of four inputs as variables. These four inputs are the four sensors (A, B, C, and D) used to construct this MLC sensor.

As shown in Figure 7, four layers of capacitive sensors can be used to construct an MLC with 15 tactels. Comparing this to a traditional array layout using rows and columns, where a 15 tactel sensor would require 15 individual capacitors, the system used in this work has an advantage as the number of tactels increased. A mathematical equation has been developed that relates the number of tactels to the number of sensors. Adding the pressure sensor layer has an additional tactel, therefore, it became 16 tactels.

For square-shape sensors, Equation (1) relates the number of tactels, *f*(*n*) (touch location) as a function of the array size, *n*.

(1) $$f\left(n\right)=\frac{3}{2}n*\left(\frac{3}{2}n+2\right)+1$$

where *n* is the sensors array of *n* × *n* (*n* must be an even number)

This equation can be simplified, as follows:

(2) $$f\left(n\right)=\frac{9}{4}n^{2}+3n+1$$

For example, using Equation (2), this example of a 2 × 2 array will result in a total of 16 tactels, a 4 × 4 array will generate 49 tactels, 6 × 6 will yield 100 tactels, and so on.

If an array is not a symmetrical array, such that the array is *m* × *n*, Equation (2) will be modified into Equation (3) that relates the number of tactels, *f*(*n*) (touch location) to the array size (*m* × *n*)

(3) $$f\left(m,n\right)=\frac{9}{4}mn+\frac{3\left(m+n\right)}{2}+1$$

where *m* is the number of sensors in horizontal, and *n* is the number of sensors number in vertical (both have to be even numbers). For example, a 2 × 4 array, will generate 28 tactels.

**Figure 10:**
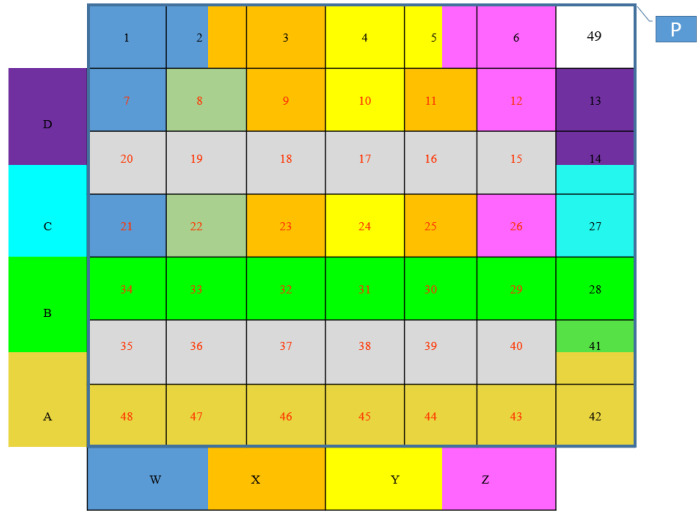
The 4 ×4 sensors 2D array with overlapping arrangement of sensors A, B, C, D, W, X, Y, and Z, plus pressure sensor (P). Each color denotes the corresponding sensors in each tactel.

As in Equations (2) and (3), the number of tactels can be increased by increasing the number of sensors from the 2 × 2 arrangement as discussed in this chapter to, for example, a 2 × 4, 4 × 4, and a 6 × 6, arrangement. The mapping of the MLC sensor can be extended to more tactels by extending the number of digital variables that correspond to the array size, as shown in Figure 10, where a 4 × 4 arrangement leads to a total of eight overlapping sensors (eight input variables, A, B, C, D, W, X, Y, and Z) that can have 48 tactels, by adding the P layer, there is total of 49 tactels for the multimodal sensor.

The MLC sensor has been developed with the number of tactels determined by the number of overlapping capacitive sensors, and it can detect touch and identify the location (tactel) of the touch.

There have been limited designs of DE keyboards developed [21]. An example is Xu’s stretch rubber keyboard [22]; however, only this proposed MLC sensor allows a truly flexible, conformal, and stretchable keyboard to be realized.

**Figure 11:**
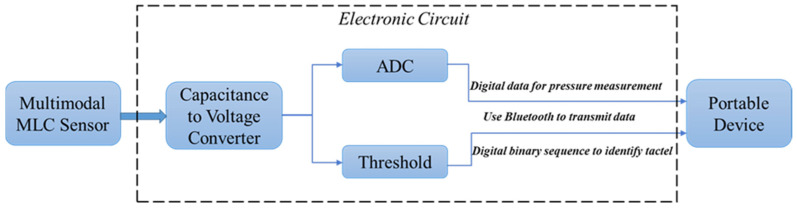
The proposed portable wireless system setup for future development for applications as wearables or as a commercial product.

In the future, it will be beneficial to develop an embedded system that can be integrated with the multimodal sensor for applications as wearables, as illustrated in Figure 11.

## 6. Conclusions

In this study, a multimodal DE force and touch sensor was proposed and demonstrated. The multimodal sensor demonstrated here is made by combining three layers (P, MLC, and substrate layers), and has 16 tactels. Using this unique arrangement, only five electrical interconnects are required compared to the traditional eight interconnects (four rows and four columns).

This multimodal sensor was able to measure the pressure and quickly identify the tactel for any single point load. The detection at all locations worked well for all the different forces applied within the 10 N range. Adaptive thresholding is required for sensors P, A, B, C, and D to generate the associated digital sequence representing the tactel location. The work presented in this study has provided a framework where the use of DE as a capacitive sensor, coupled with the clever arrangement of overlapping configuration, allows the creation of a multimodal sensor that is easy to fabricate without the need for precision fabrication techniques. However, there are some limitations of the multimodal DE sensor, such as the multi-modular sensor structure sensor elements array within the MCL layers must be an even number. Also, if the DE piece gets too large, the capacitance will be large, and it increases the time required to read the signal. For the multi-touch function, it would require developing a complex algorithm to decipher multiple tactels being pressed simultaneously.

Compared with existing sensors, the advantage of the multimodal DE sensor presented here is the reduction in the number of sensor strips and connection wires, without a corresponding reduction in touchpoint detection. By using a compliant substrate with optimal thickness, it had a higher sensitivity while remaining soft and flexible. The required wiring within the multimodal sensor is minimal compared to those that are traditionally made by using rows and columns to access each sensing element. The multimodal sensor developed here shows promise for various applications in soft robotics and wearable devices requiring a solution for identifying the different locations and pressure of contacts.

## Figures and Tables

**Table 1:** Truth table showing digital outputs representing each tactel.

	Sensors	P	A	B	C	D
Tactel #						
**1**		1	0	0	1	0
**2**		1	0	0	1	1
**3**		1	0	0	0	1
**4**		1	1	0	1	0
**5**		1	1	0	1	1
**6**		1	1	0	0	1
**7**		1	1	0	0	0
**8**		1	1	1	0	0
**9**		1	1	1	0	1
**10**		1	1	1	1	1
**11**		1	1	1	1	0
**12**		1	0	1	1	0
**13**		1	0	1	1	1
**14**		1	0	1	0	1
**15**		1	0	1	0	0
**16**		1	0	0	0	0
